# Systematic review of melatonin in cerebral ischemia-reperfusion injury: critical role and therapeutic opportunities

**DOI:** 10.3389/fphar.2024.1356112

**Published:** 2024-02-05

**Authors:** Chenguang Zhang, Yumei Ma, Yating Zhao, Na Guo, Chen Han, Qian Wu, Changqing Mu, Yue Zhang, Shutong Tan, Jian Zhang, Xu Liu

**Affiliations:** ^1^ Department of Neurology, First Affiliated Hospital of China Medical University, Shenyang, Liaoning, China; ^2^ Department of Cell Biology, Key Laboratory of Cell Biology, Ministry of Public Health, Shenyang, Liaoning, China; ^3^ Key Laboratory of Medical Cell Biology, Ministry of Education, China Medical University, Shenyang, Liaoning, China

**Keywords:** melatonin, cerebral ischemia-reperfusion injury, ischemic stroke, neuroprotection, systematic review

## Abstract

Cerebral ischemia-reperfusion (I/R) injury is the predominant causes for the poor prognosis of ischemic stroke patients after reperfusion therapy. Currently, potent therapeutic interventions for cerebral I/R injury are still very limited. Melatonin, an endogenous hormone, was found to be valid in preventing I/R injury in a variety of organs. However, a systematic review covering all neuroprotective effects of melatonin in cerebral I/R injury has not been reported yet. Thus, we perform a comprehensive overview of the influence of melatonin on cerebral I/R injury by collecting all available literature exploring the latent effect of melatonin on cerebral I/R injury as well as ischemic stroke. In this systematic review, we outline the extensive scientific studies and summarize the beneficial functions of melatonin, including reducing infarct volume, decreasing brain edema, improving neurological functions and attenuating blood-brain barrier breakdown, as well as its key protective mechanisms on almost every aspect of cerebral I/R injury, including inhibiting oxidative stress, neuroinflammation, apoptosis, excessive autophagy, glutamate excitotoxicity and mitochondrial dysfunction. Subsequently, we also review the predictive and therapeutic implications of melatonin on ischemic stroke reported in clinical studies. We hope that our systematic review can provide the most comprehensive introduction of current advancements on melatonin in cerebral I/R injury and new insights into personalized diagnosis and treatment of ischemic stroke.

## 1 Introduction

Ischemic stroke is one of the main causes of mortality and the leading cause of adult disability worldwide. It affects the lives of millions of patients and imposes a heavy financial burden on the society (Regenhardt et al., 2018; Zhou et al., 2018; Datta et al., 2020). According to estimates in 2020, the average costs of hospitalization on ischemic stroke per person was amounted to $18,154 and the 3-year follow-up cost was $44,347 in the United States (Yousufuddin et al., 2020). Currently, therapeutic options for ischemic stroke are still very limited and the only drug authorized by FDA is recombinant tissue plasminogen activator (t-PA), which can accelerate clot dissolution and blood reperfusion. However, administration of t-PA is only suitable for about 5% of ischemic stroke patients due to the narrow therapeutic window (Zhou et al., 2018; Powers et al., 2019). Moreover, it may paradoxically result in cerebral I/R injury owing to blood flow restoration, which can lead to reactive oxygen free radical accumulation and thus cause brain edema, neurological deficits as well as individual death through a series of mechanisms, including oxidative stress, neuroinflammation, apoptosis, excessive autophagy, glutamate excitotoxicity, and mitochondrial dysfunction (Duehrkop and Rieben, 2014; Sarmah et al., 2018; Datta et al., 2020). Nowadays, a valid treatment for cerebral I/R injury has not been determined. Despite several substances have shown neuroprotective effects on cerebral I/R injury models, consistent results are not be obtained in further clinical trials. Thus, more potentially effective substances are being actively studied, among which melatonin is the most concerned.

Melatonin (N-acetyl-5-methoxytrytamine), an amine hormone discovered by Aaron Lerner half a century ago, is mostly secreted by the pineal gland and exists in extra-pineal sites including the retina, gastrointestinal tract, and bone marrow as well (Zawilska JolantaB. et al., 2009; Samantaray et al., 2009; Venegas et al., 2012). The timing of melatonin secretion adapts to the light-dark cycle (Zawilska, Skene, and Arendt, 2009a). Therefore, it is originally considered to be responsible for controlling circadian rhythms, including sleep-wake rhythm, neuroendocrine rhythm as well as seasonal response, by providing night information and mediating dark signals (Claustrat, Brun, and Chazot, 2005). Moreover, melatonin is involved in lowering core body temperature at night and promotes sleep tendency, whereas its secretion is disrupted upon exposure to light, thus causing wakefulness (Akerstedt et al., 1979). Additionally, melatonin plays important physiological roles in multiple other systems: cardiovascular system, respiratory system, reproductive system, immune system, and endocrine system, etc., For example, recent studies have indicated that melatonin could take participate in the regulation of blood pressure and heart rate via endothelium-dependent vasodilation and sympathovagal autonomic modulation (Girouard et al., 2001; Baker and Kimpinski, 2018). Besides, when combing with melatonin receptors on pancreatic islets, melatonin could induce the production of insulin growth factors, and subsequently promote insulin receptor tyrosine phosphorylation, thereby modulating insulin sensitivity and glucose homeostasis (Sharma et al., 2015).

To date, melatonin is thought to exert various physiological effects primarily through its action on two specific membrane receptors, MT1 and MT2. As members of the seven-transmembrane G protein-coupled receptor family, these two MT receptors are widely distributed throughout the central nervous system, including cerebral cortex, hippocampus, cerebellum, midbrain and especially hypothalamus and suprachiasmatic nucleus (Ekmekcioglu, 2006; Ng et al., 2017). Moreover, MT1 receptors are more abundant than MT2 receptors in most brain regions. Recent researches have demonstrated that in the various CNS regions, melatonin may exert multiple neuroprotective effects by direct binding to MT1 and MT2 receptors. In mouse primary neurons, Liu et al. observed that melatonin supplementation could activate Akt/GSK-3β/CRMP-2 signaling by acting on MT2 receptors, subsequently enhance excitatory synaptic transmission and thus promote functional and synaptic formation (Liu et al., 2015). Similarly, in APP/PS1 transgenic mice, melatonin administration could reverse mitochondrial dysfunction and decrease abnormal Aβ deposition in the cortex, striatum, and hippocampus brain region via MT2 receptor signaling, eventually improving cognitive and behavior deficits (Dragicevic et al., 2011). Additionally, in a transgenic mouse model of Huntington’s disease (HD), MT1 receptor levels in mice brain were much lower compared with wild-type controls. However, melatonin treatment could inhibit mutant huntingtin-induced caspase-3 activation and preserve MT1 receptor expression. Furthermore, melatonin would delay disease onset and mortality of HD mice, which was dependent on the presence and activation of MT1 receptor (Wang et al., 2011).

More importantly, emerging studies have confirmed that melatonin also plays an important effect in preventing I/R injury in a variety of organs including heart, liver, and brain (Tai et al., 2010; Kang et al., 2011; Zhang et al., 2019). Zhang et al. observed that melatonin administration contributed to improve mitochondrial fusion/mitophagy through inhibiting I/R-mediated Optic atrophy 1 (OPA1) downregulation, thereby maintaining myocardial function and cardiomyocyte viability in myocardial I/R injury (Zhang et al., 2019). Additionally, Kang et al. confirmed that melatonin could protect against hepatic I/R injury via mitigating Toll-like receptor 3 (TLR3) and Toll-like receptor 4 (TLR4) overexpression, which further inhibited TLR-mediated downstream inflammatory signaling cascades, including myeloid differentiation primary response 88 (MyD88)-dependent and toll-receptor-associated activator of interferon (TRIF)-dependent pathways (Kang, Koh, and Lee, 2011). As for cerebral I/R injury, Tai et al. demonstrated that melatonin reduced endothelial damage and preserved blood-brain barrier (BBB) integrity via attenuating matrix metalloproteinase-9 (MMP-9) protein expression and activity, thereby exerting neuroprotective effects (Tai et al., 2010). Nowadays, growing studies have demonstrated that melatonin-induced protective effects exert an important role in the brain. Thus, melatonin is expected to become a latent therapeutic agent in brain I/R injury. However, almost no systematic review that covering all protective effects of melatonin in cerebral I/R injury has been reported. Based on existing evidence, we conduct this comprehensive review aiming to study the neuroprotective effect of melatonin and the underlying molecular mechanisms, and explore the latent clinical application prospects of melatonin in cerebral I/R injury together with ischemic stroke.

## 2 Article search strategy process and study selection

**FIGURE 1:**
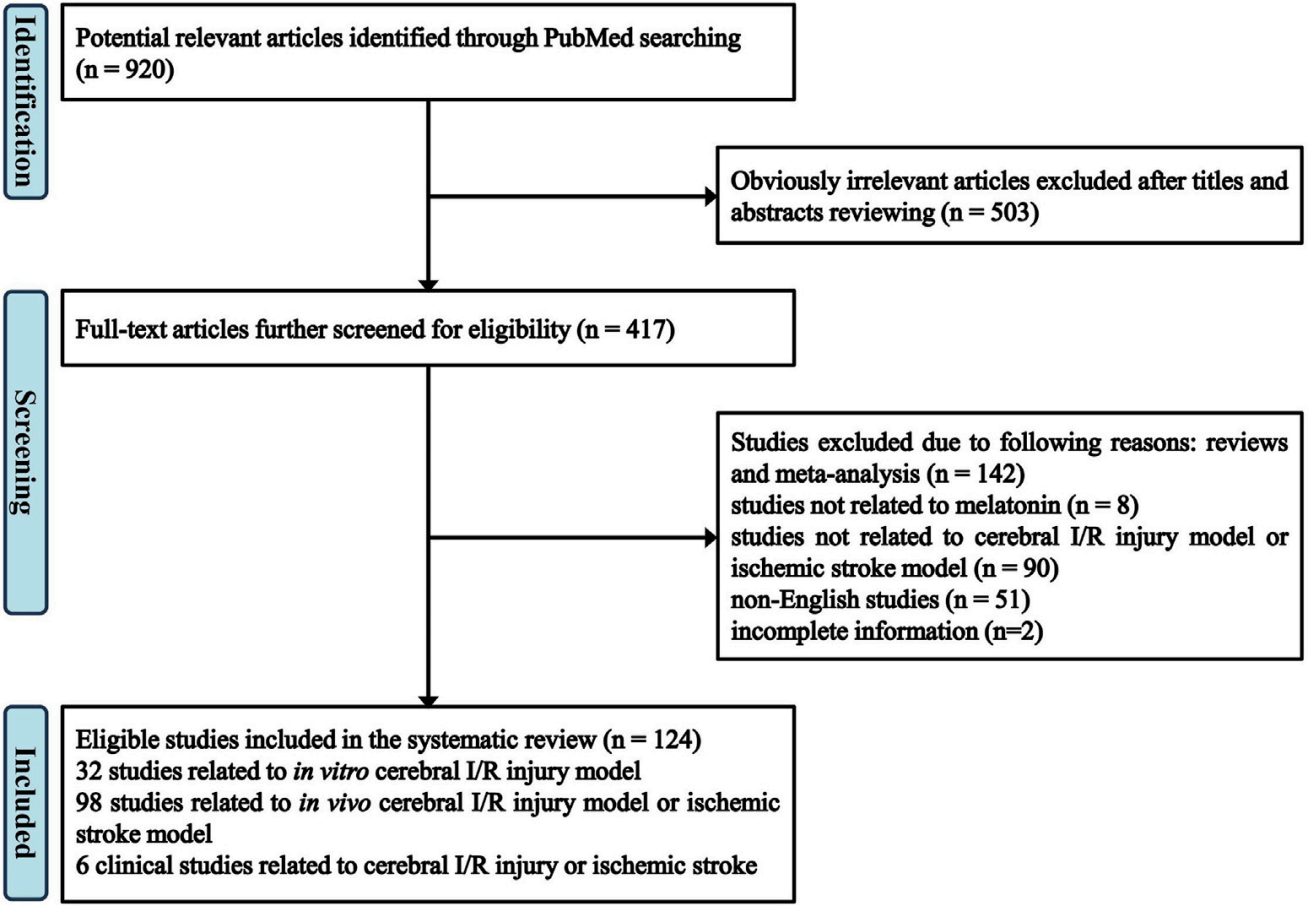
Flow diagram of study identification and selection.

A total of 920 articles are identified in PubMed database by using the following terms: melatonin and cerebral ischemia/brain ischemia/cerebral infarction/brain infarction/stroke/ischemia-reperfusion. Among them, by screening the titles and abstracts, 503 apparently irrelevant articles are excluded. 293 articles are further removed after reviewing the full-text articles for eligibility, among which 142 articles are reviews and meta-analyses, 8 articles are not related to melatonin, 90 articles are not related to cerebral I/R injury model or ischemic stroke model, including traumatic brain injury (TBI), neurodegenerative diseases, neonatal encephalopathy, 51 articles are not published in English, and 2 articles are with incomplete information. At last, 124 eligible studies are included in the review. Specifically, 32 studies are focusing on *in vitro* cerebral I/R injury model (Table 1), 98 studies on *in vivo* cerebral I/R injury model or ischemic stroke model (Table 2), 6 clinical studies are related to cerebral I/R injury or ischemic stroke. The flow of screening process is shown in Figure 1.

**TABLE 1:** Protective effects of melatonin on *in vitro* cerebral I/R injury model.

Time	Dose	Effect	Mechanism	References
N2a cell line				
24 h before HR	10, 20 µM	reduced cell death	alleviated mitochondrial dysfunction	Wei et al. (2019)
2 h before HR	1, 5, 10, 20 µM	increased cell viability	anti-oxidative stress, anti-apoptosis	Xing et al. (2019)
30 min after OGD	100, 200, 400 mM	reduced cell death	anti-inflammation	Liu, Ran, et al. (2019)
immediately before reoxygenation	1, 10, 100 µM	increased cell viability	anti-apoptosis	Duan et al. (2006)
immediately before reoxygenation	12.5, 25, 50, 100 nM	reduced cell death	anti-apoptosis	Ran et al. (2021)
immediately before reoxygenation	50 µM	reduced cell death	anti-oxidative stress, anti-apoptosis, alleviated mitochondrial dysfunction	Chen et al. (2021)
SH-SY5Y cell line				
immediately before OGD	0.01, 0.1, 1 mM	reduced cell death	anti-oxidative stress, alleviated mitochondrial dysfunction	Pei and Cheung, (2003)
immediately before reoxygenation	0.01, 0.1, 1, 10 mM	reduced cell death, increased cell viability	anti-oxidative stress, anti-inflammation, anti-apoptosis, inhibited autophagy	Zhi et al. (2020)
PC12 cell line				
30 min before OGD	50 µM	reduced cell death	anti-oxidative stress, anti-inflammation, anti-apoptosis, inhibit autophagy	Yang, Zang, et al. (2020)
HT22 cell line				
4 h before OGD	25, 50, 100 µM	increased cell viability	alleviated mitochondrial dysfunction	Liu, Cao, Gao, Li, Xia, et al. (2021)
immediately before OGD	50 µM	reduced cell death	-	Luchetti et al. (2022)
immediately before reoxygenation	25, 50, 100 µM	reduced cell death	anti-oxidative stress, anti-apoptosis	Liu, Cao, Gao, Li, Zeng, et al. (2021)
immediately before reoxygenation	50 µM	reduced cell death	anti-oxidative stress, alleviated mitochondrial dysfunction	Nasoni et al. (2021)
BV-2 cell line				
24 h before HR	1, 5, 10, 30, 100, 300 µM	increased cell viability	anti-inflammation	Azedi et al. (2019)
primary cortical neurons				
30 min before OGD	10, 20, 50, 100, 500 µM	reduced cell death	anti-apoptosis	Lin et al. (2018)
immediately before OGD	1, 5, 10 µM	reduced cell death	anti-oxidative stress, anti-inflammation	Tai et al. (2011)
immediately before OGD	1, 10, 30, 100 µM	reduced cell death	anti-apoptosis	Wang et al. (2009)
immediately before reoxygenation	6.25, 12.5, 25, 50 nM	reduced cell death	anti-apoptosis	Ran et al. (2021)
primary cerebellar granule neurons				
immediately before reoxygenation	0.001, 0.1, 10 µM	reduced cell death	anti-apoptosis	Han et al. (2006)
primary striatal neurons				
immediately before OGD and reoxygenation	100 µM	reduced cell death	anti-apoptosis	Andrabi et al. (2004)
hippocampal slice cultures				
30 min before OGD	1, 3, 10, 30 µM	reduced cell death	inhibited glutamate excitotoxicity	Patino et al. (2016)
30 min before OGD	30, 100 µM	reduced cell death	anti-oxidative stress	Gasparova et al. (2006)
immediately before OGD	1, 3, 10, 25, 50, 100 µM	reduced cell death	anti-apoptosis	Carloni et al. (2018)
immediately before OGD	50 µM	reduced cell death	-	Luchetti et al. (2022)
immediately before HR	0.1, 1, 10, 100 µM	reduced cell death	anti-oxidative stress	Uchida et al. (2004)
0, 1, 2 h after OGD	0.1, 1, 10 µM	reduced cell death	anti-oxidative stress	Parada et al. (2014)
30 min before and after OGD/R	1, 10, 100, 1,000 nM	reduced cell death	anti-oxidative stress, inhibited glutamate excitotoxicity	Buendia et al. (2015)

HR: hypoxia/reoxygenation; OGD/R: oxygen and glucose deprivation/reoxygenation.

**TABLE 2:** Protective effects of melatonin on *in vivo* cerebral I/R injury model or ischemic stroke model.

Route	Time	Dose (mg/kg)	Effect	Mechanism	References
Sprague-Dawley rats					
i.p.	3 h before MCAO	50	reduced brain infarct volume, improved neurological function	anti-oxidative stress, anti-apoptosis, alleviated mitochondrial dysfunction	Yip et al. (2021)
i.p.	30 min before MCAO	5	reduced brain infarct volume, improved neurological function	anti-oxidative stress, anti-inflammation, anti-apoptosis, inhibited glutamate excitotoxicity	Pei et al. (2002), Pei and Cheung, 2004; Koh (2008a), Koh, 2008b; Koh (2008c), Koh, 2008d; Koh (2008e), Koh (2011), Koh, 2012a; Koh, 2012b; Li et al., 2014; Shah et al., 2018; Shah et al., 2019; Ling et al., 2020
i.p.	30 min before MCAO	10	reduced brain infarct volume, improved neurological function	anti-inflammation, anti-apoptosis	Liu and Cheung, 2013; Ma et al., 2013; Xu and Cheung, 2023
i.p.	30 min before MCAO	15	reduced brain infarct volume	anti-oxidative stress	Pei et al. (2002), Pei et al. (2002)
i.p.	30 min before MCAO	50	reduced brain infarct volume, preserved BBB integrity	anti-oxidative stress	Pei et al. (2002), Pei et al. (2003)
i.p.	30 min after MCAO	10, 20, 40	improved neurological function	anti-apoptosis	Chen et al. (2022)
i.p.	1 h after MCAO	5	reduced brain infarct volume	-	Pei et al., (2003)
i.p.	5 min before reperfusion	10	reduced brain infarct volume and brain edema, improved neurological function	anti-oxidative stress, anti-apoptosis	Gupta et al. (2004)
i.p.	immediately before reperfusion	10	reduced brain infarct volume	anti-apoptosis	Sung et al. (2009)
i.p.	30 min after reperfusion	50	reduced brain infarct volume, improved neurological function	anti-oxidative stress, anti-apoptosis, alleviated mitochondrial dysfunction	Chen et al. (2021)
i.p.	1.5, 24, 48 h after reperfusion	20,50	educed brain infarct volume, improved neurological function	anti-oxidative stress, anti-inflammation	Lin et al. (2019)
i.p.	6, 24, 48 h after reperfusion	30	reduced brain infarct volume, improved neurological function	anti-oxidative stress, anti-apoptosis, alleviated mitochondrial dysfunction	Chen et al. (2021)
i.p.	once daily before MCAO	10	reduced brain infarct volume and brain edema, improved neurological function	inhibited autophagy	Feng et al. (2017)
i.p.	once daily after MCAO	5, 10	reduced brain infarct volume, improved neurological function	anti-oxidative stress, anti-inflammation, anti-apoptosis, inhibited autophagy	Nair et al., 2011; Zheng et al., 2014; Hao et al., 2021
i.p.	twice daily after PT	50	preserved BBB integrity	anti-inflammation	Jang et al. (2012)
i.p.	30 min before MCAO and 30 min after reperfusion	5	reduced brain infarct volume and brain edema, improved neurological function	anti-oxidative stress, anti-inflammation	Zhao et al., 2018; Zhao et al., 2019a
i.p.	15 min before MCAO and 6, 12 h after reperfusion	10	reduced brain infarct volume	anti-oxidative stress, anti-apoptosis	Sun et al. (2002)
i.v.	immediately before reperfusion	5	reduced brain infarct volume and brain edema, improved neurological function	anti-inflammation, anti-apoptosis, inhibited glutamate excitotoxicity	Lee et al., 2004; Lee et al., 2007; Hung et al., 2008; Chen et al., 2009; Juan et al., 2014; Lin et al., 2018
i.v.	immediately before reperfusion	15, 50	reduced brain infarct volume, improved neurological function	anti-oxidative stress, anti-inflammation	Tai et al. (2011)
s.c.	30 min before MCAO	4	-	anti-oxidative stress	Li et al. (1997)
s.c.	15 min before and 6, 12 h after MCAO	2.5, 5, 10	reduced brain infarct volume	anti-apoptosis	Ling et al. (1999)
Wistar rats					
i.p.	24 h before MCAO	10	reduced brain infarct volume	anti-inflammation, anti-apoptosis	Rancan et al. (2018)
i.p.	1 h before PT	100	reduced brain infarct volume	anti-oxidative stress	Matejovska et al. (2008)
i.p.	30 min before and immediately before MCAO	10	improved neurological function	anti-oxidative stress, anti-apoptosis	Ahmad et al. (2011)
i.p.	immediately before reperfusion	10	reduced brain infarct volume, improved neurological function	anti-inflammation, anti-apoptosis	Yawoot et al. (2023)
i.p.	10 min after PT	100	improved neurological function	anti-oxidative stress	Deykun et al. (2011)
i.p.	1h after reperfusion and once daily after MCAO	10	reduced brain infarct volume	anti-apoptosis	Yilmaz et al. (2023)
i.p.	0, 1.5 h after MCAO and 0, 1 h after reperfusion	20	reduced brain infarct volume, improved neurological function	anti-oxidative stress	Gupta et al. (2002)
i.p.	0, 1 h after MCAO and reperfusion	10, 20, 40	reduced brain infarct volume, improved neurological function	anti-oxidative stress	Sinha et al. (2001)
i.p.	once daily after MCAO	10	reduced brain infarct volume, improved neurological function	anti-oxidative stress, anti-inflammation, anti-apoptosis	Rancan et al. (2018)
i.p.	immediately before MCAO and reperfusion	10	reduced brain infarct volume	anti-apoptosis	Ma et al. (2013)
i.p.	immediately before and once daily after reperfusion	3, 10	reduced brain infarct volume, improved neurological function	anti-inflammation	Yawoot et al. (2022)
i.a.	at the end of reperfusion	4	reduced brain infarct volume, improved neurological function	anti-inflammation	Azedi et al. (2019)
p.o.	once daily after reperfusion	10	reduced brain infarct volume	anti-oxidative stress, anti-inflammation	Saleh et al. (2020)
p.o.	24 h before and 24 h after MCAO	10	reduced brain infarct volume	anti-inflammation, anti-apoptosis	Paredes et al. (2015)
p.o.	1 h before and 24 h after MCAO	6	reduced brain edema	anti-oxidative stress	Kondoh et al., 2002; Torii et al., 2004
p.o.	1 h before and once daily after MCAO	0.87–0.1	reduced brain infarct volume	-	Borlongan et al. (2000)
Charles Foster rats					
i.p.	30 min before and 1, 2 h after MCAO	1, 3, 5, 7, 9	reduced brain infarct volume and brain edema, improved neurological function	-	Bhattacharya et al. (2014)
C57BL/6j mice					
i.p.	before MCAO	10	reduced brain infarct volume, brain edema	anti-oxidative stress, anti-apoptosis	Zang et al. (2020)
i.p.	5 min after PT	1	reduced brain infarct volume	anti-oxidative stress, anti-apoptosis, inhibited glutamate excitotoxicity	Buendia et al. (2015)
i.p.	0, 24, 48 h after MCAO	5, 10, 20	reduced brain infarct volume, improved neurological function	anti-apoptosis	Ran et al. (2021)
i.p.	immediately before reperfusion	4	reduced brain infarct volume and brain edema, preserved BBB integrity	anti-apoptosis	Kilic et al., 2004; Kilic et al. 2005a; Kilic et al. 2005b; Kilic et al. 2013; Kilic et al. 2017
i.p.	immediately before reperfusion	5	reduced brain infarct volume, improved neurological function, preserved BBB integrity	anti-oxidative stress	Lee et al., 2005; Chen, Chen, et al., 2006; Chen et al., 2006
i.p.	immediately before reperfusion	15	reduced brain infarct volume, improved neurological function, preserved BBB integrity	anti-oxidative stress, anti-inflammation	Parada et al., 2014; Chen, Sun, et al., 2022
i.p.	once daily after MCAO	4	improved neurological function	anti-inflammation	Kilic et al. (2021)
i.p.	1 h before and 30 min after MCAO	10	reduced brain infarct volume	anti-apoptosis	Wang et al. (2009)
i.p.	30 min before and 24, 48 h after PT	15	reduced brain infarct volume	anti-inflammation	Zou et al. (2004)
i.p.	immediately before MCAO and reperfusion	4	reduced brain infarct volume	anti-apoptosis	Kilic et al. (2004)
i.p.	immediately before MCAO and reperfusion	5, 10	reduced brain infarct volume and brain edema, improved neurological function	anti-oxidative stress, anti-apoptosis, alleviated mitochondrial dysfunction	Yang et al., 2015; Liu et al., 2021
i.p.	immediately before MCAO and reperfusion	10	reduced brain infarct volume, improved neurological function	anti-oxidative stress, anti-apoptosis	Liu et al. (2021)
i.p.	immediately before MCAO and reperfusion	20	reduced brain infarct volume, improved neurological function	anti-apoptosis	Liu et al. (2019)
i.p.	immediately before and once daily after MCAO	20	reduced brain infarct volume, improved neurological function	anti-inflammation	Li et al. (2023)
i.p.	3 days before and once daily after MCAO	20, 50	reduced brain infarct volume	anti-oxidative stress, anti-inflammation	Chen et al. (2020)
i.v.	1 h after MCAO	5	reduced brain infarct volume	anti-oxidative stress	Tai et al. (2010)
i.v.	0, 24 h after reperfusion	20	reduced brain infarct volume, improved neurological function	anti-inflammation	Liu et al. (2019)
p.o.	once daily after MCAO	4	improved neurological function	-	Kilic et al. (2008)
Balb/c mice					
i.p.	1 min after reperfusion	4	reduced brain infarct volume and brain edema, improved neurological function	anti-apoptosis	Beker et al. (2015)
ICR mice					
i.p.	once daily after MCAO	5, 10	reduced brain infarct volume, improved neurological function	anti-oxidative stress, anti-inflammation	Chern et al. (2012)
129/C57BL mice					
i.p.	30 min before and 24, 48 h after PT	15	reduced brain infarct volume	anti-inflammation	Zou et al. (2006)
B6CBA mice					
i.p.	1 h after and once daily after MCAO	15	reduced brain infarct volume	anti-inflammation	Suofu et al. (2023)

i.p.: intraperitoneal; i.v.: intravenous; p.o.: peros; s.c.: subcutaneous; i.a.: intra-arterial; MCAO/R: middle cerebral artery occlusion/reperfusion; PT: photothrombotic stroke; BBB: blood-brain barrier.

## 3 Protective effects of melatonin in experimental I/R injury models

During ischemic stroke, blood supply for the brain is interrupted, which can cause irretrievable tissue damage. Ischemic tissue injury is commonly attributed to tissue hypoxia and the subsequent cellular ATP depletion, which may in turn cause death of neurons and glia and then lead to the brain tissue infarction as well as neurological deficits. After reperfusion, the generation of reactive oxygen species (ROS) is at an accelerated rate in tissues (Granger and Kvietys, 2015). The central nervous system (CNS) is particularly sensitive to ROS because it contains high concentrations of substances that promote oxidative processes, including iron, ascorbic acid, and polyunsaturated fatty acids, while low amounts of endogenous antioxidants (Watson et al., 2016). ROS accumulation can cause reperfusion injury, including programmed cell death and necrosis in the brain through triggering a cascade characterized by ionic imbalance, mitochondrial disturbances, oxidative stress and endoplasmic reticulum (ER) stress (Murphy and Steenbergen, 2008; Bagheri et al., 2016; Lin et al., 2018). Thus, protecting CNS cells from I/R injury is considered as an important therapeutic strategy for ischemic stroke treatment. For investigations of cerebral I/R injury, the preferred *in vivo* model is middle cerebral artery occlusion (MCAO), including transient MCAO and permanent MCAO (Lemmerman et al., 2022). Besides, neurons and glial cells subjected to oxygen-glucose deprivation/reoxygenation (OGD/R) are common *in vitro* models, allowing us to investigate the specific feature of different cell types.

As a free radical scavenger and antioxidant, melatonin has been inferred to exert a neuroprotective effect in cerebral I/R injury. Up till now, large amounts of studies have indicated that treatment with melatonin before and after OGD can exert neuroprotective effects in CNS cells. Detailed characteristics of included studies are shown in Table 1 (Vlkolinsky and Stolc, 1999; Pei and Cheung, 2003; Andrabi et al., 2004; Uchida et al., 2004; Duan et al., 2006; Gasparova, Stolc, and Snirc, 2006; Han et al., 2006; Wang et al., 2009; Guo et al., 2010; Tai et al., 2011; Parada et al., 2014; Buendia et al., 2015; Huang et al., 2015; Patino et al., 2016; Suofu et al., 2017; Carloni et al., 2018; Lin et al., 2018; Azedi et al., 2019; Beker et al., 2019; Liu, Ran, et al., 2019; Wei et al., 2019; Xing et al., 2019; Xiang et al., 2020; Yang, Zang, et al., 2020; Zhi et al., 2020; Liu, Cao, Gao, Li, Xia, et al., 2021; Liu, Cao, Gao, Li, Zeng, et al., 2021; Nasoni et al., 2021; Ran et al., 2021; Luchetti et al., 2022; Sun et al., 2022; Su et al., 2023). Lin et al. reported that exposure of cultured neurons obtained from the cerebral cortices of Sprague-Dawley rats to 30 min of OGD and 1, 2, 4, and 24 h of reoxygenation caused the increase of cell apoptosis, while pretreatment with melatonin (10–500 µM) before OGD/R for 30 min could promote the survival of neurons (Y.W. Lin et al., 2018). Subsequently, Yang et al. confirmed that compared with control groups, pretreatment with melatonin (50 µM) in the PC12 cell line exposed to OGD for 30 min and reoxygenation for 24 h had a lower neuronal apoptosis rate (Yang, Zang, et al., 2020). In addition, in cultured neurons from CA1 and CA3 hippocampal regions, administration of melatonin (1, 3, 10, and 30 µM) after OGD/R could lead to a decrease in OGD/R-induced neuronal injury and death compared to control groups (Patino et al., 2016). Similarly, consistent results were also observed in a study using melatonin-treated SH-SY5Y cell line models performed by Zhi et al. (Zhi et al., 2020). Apart from neuronal cells, the neuroprotective effect of melatonin has also been confirmed in glial cells. In BV-2 cell line, pretreatment with melatonin (10, 100, and 300 µM) at 24 h prior to OGD/R was found to increase the viability of cells exposed to 3 h of OGD and 3, 6, and 24 h of reoxygenation (Azedi et al., 2019).

More importantly, emerging studies have indicated that melatonin exert neuroprotective effects in stroke animals (Table 2) (Li et al., 1997; Kilic et al., 1999; Ling et al., 1999; Borlongan et al., 2000; Sinha et al., 2001; Gupta et al., 2002; Kondoh et al., 2002; Pei et al., 2002; Pei et al., 2002; Sun et al., 2002; Pe and Cheung, 2003; Pei et al., 2003; Andrabi et al., 2004; Gupta et al., 2004; Lee et al., 2004; Pei and Cheung, 2004; Torii et al., 2004; Zou et al., 2004; Kilic E. et al., 2005; Kilic U. et al., 2005; Lee et al., 2005; Chen et al., 2006; Chen et al., 2006; Zou et al., 2006; Lee et al., 2007; Koh, 2008a; Koh, 2008b; Koh, 2008c; Koh, 2008d; Koh, 2008e; Hung et al., 2008; Kilic et al., 2008; Matejovska et al., 2008; Chen et al., 2009; Sung et al., 2009; Wang et al., 2009; Tai et al., 2010; Ahmad et al., 2011; Deykun et al., 2011; Koh, 2011; Nair et al., 2011; Tai et al., 2011; Koh, 2012a; Koh, 2012b; Chern et al., 2012; Jang et al., 2012; Kilic et al., 2012; Kilic et al., 2013; Liu and Cheung, 2013; Ma et al., 2013; Bhattacharya et al., 2014; Juan et al., 2014; Li et al., 2014; Zheng et al., 2014; Beker et al., 2015; Buendia et al., 2015; Paredes et al., 2015; Yang et al., 2015; Chumboatong et al., 2017; Feng et al., 2017; Kilic et al., 2017; Lin et al., 2018; Rancan et al., 2018; Shah et al., 2018; Zhao et al., 2018; Azedi et al., 2019; Zhao et al., 2019a; Lin et al., 2019; Liu, Chen, et al., 2019; Shah et al., 2019; Wei et al., 2019; Chen et al., 2020; Chumboatong et al., 2020; Ling et al., 2020; Saleh et al., 2020; Yang et al., 2020; Zang et al., 2020; Chen et al., 2021; Hao et al., 2021; Kilic et al., 2021; Liu et al., 2021; Liu et al., 2021; Ran et al., 2021; Shao et al., 2021; Yip et al., 2021; Cambiaghi, Cherchi, and Comai, 2022; Chen et al., 2022; Chen et al., 2022; Kilic et al., 2022; Yawoot et al., 2022; Li et al., 2023; Su et al., 2023; Suofu et al., 2023; Xu and Cheung, 2023; Yawoot et al., 2023; Yilmaz et al., 2023). For example, mice that exposed to focal cerebral ischemia for 30 min and reperfusion for 72 h exhibited brain damage and neurological dysfunction, while intraperitoneal administration of melatonin (4 mg/kg) prior to MCAO could decrease the neurological deficits by maintaining the integrity of BBB and reducing brain edema formation as well as infarct volume (Kilic et al., 2017). Besides, in a rat MCAO/R model, treatment with melatonin (5 mg/kg) intraperitoneally at the 30 min after I/R significantly reduced cerebral infarct volumes, promoted myelination as well as alleviated white matter damage (Zhao et al., 2019a). Furthermore, Liu et al. demonstrated that in C57BL/6J mice receiving MCAO/R, not only the cerebral infarct size was reduced, but the sensorimotor function was also improved after intraperitoneal injection of melatonin (Liu et al., 2019).

## 4 The mechanism of melatonin’s neuroprotective effect in cerebral I/R injury

**FIGURE 2:**
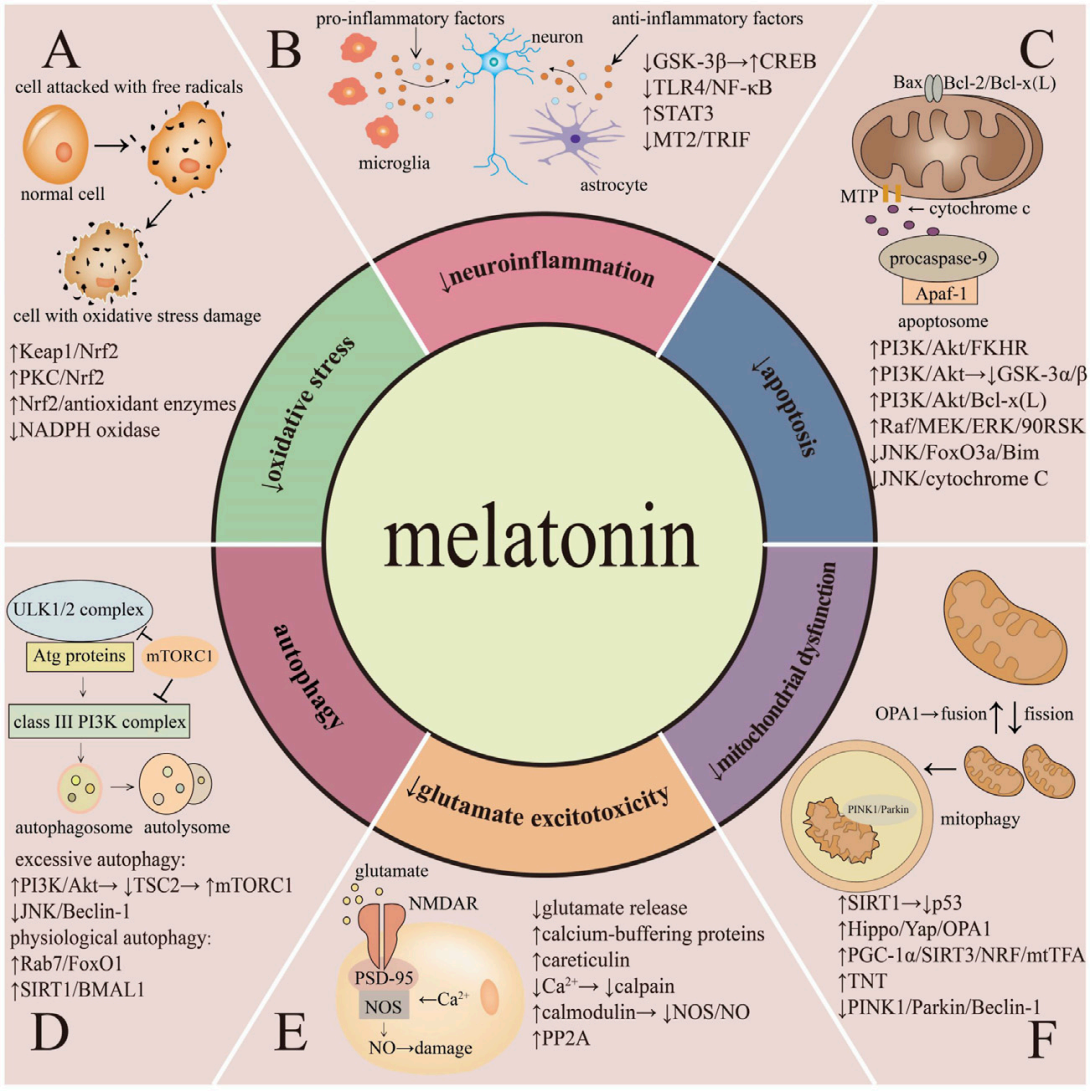
Protective mechanisms of melatonin in cerebral ischemia-reperfusion injury.

Melatonin could exert neuroprotective effects through various mechanisms in cerebral I/R injury (Figure 2).

### 4.1 Oxidative stress

Oxidative stress is characterized by excessive ROS and reactive nitrogen species (RNS) production under harmful stimuli. During cerebral I/R injury, ROS overproduction causes immense damage to brain (Manzanero et al., 2013). Compelling evidence suggests that, as a powerful scavenger against ROS/RNS, melatonin can inhibit oxidative stress in cerebral I/R injury via multiple mechanisms, among which nuclear factor E2-related factor-2 (Nrf2) occupies a vital position.

Melatonin can exert neuroprotective effects based on the upregulation of Nrf2 and Nrf2-related antioxidants during cerebral oxidative stress injury. Specifically, melatonin could reduce the ubiquitination of Nrf2 via modifying Kelch-like ECH-associated protein 1 (Keap1) cysteine residues, thereby reducing Nrf2 degradation by proteasome and enhancing Nrf2 phosphorylation and nuclear translocation to the nucleus (Zhao et al., 2018). In addition, melatonin can promote Ca^2+^ influx and induce phosphorylation of protein kinase C (PKC), an upstream mediator of Nrf2, which further leads to the downstream Nrf2 nuclear translocation (Romero et al., 2010). Moreover, melatonin can directly serve as a proteasome inhibitor to decrease the degradation of Nrf2 (Vriend and Reiter, 2014).

Nrf2, an antioxidative transcription factor, can positively regulate the expression of a series of antioxidant response element (ARE)-dependent genes. Melatonin induced Nrf2 translocation and upregulation could increase antioxidant enzymes expression levels that comprise glutathione (GSH), glutathione s-transferase (GST), superoxide dismutase (SOD), catalase (CAT), heme oxygenase-1(HO-1) and glutathione peroxidase (GPx), etc (Rodriguez et al., 2004; Ma, 2013; Zhang et al., 2013). These upregulated enzymes effectively scavenge free radicals as well as related reactants and ultimately reduce the cerebral damage during the oxidative stress process (Deng et al., 2015; Liu et al., 2019).

As shown in a former study by Parada et al., in hippocampal cultures exposed to OGD/R, administration with melatonin could induce the Nrf2 nuclear accumulation as well as the combination of Nrf2 and ARE sequences within HO-1 promoter, thereby elevating HO-1 expression and decreasing the production of OGD-induced ROS to basal levels (Parada et al., 2014). During heme metabolic process, HO-1 can reduce the ROS by degrading free heme. Meanwhile, the by-products of heme catabolism such as carbon monoxide (CO) and biliverdin (BV)/bilirubin (BR) can not only directly scavenge OGD-induced ROS production, but also inhibit NADPH oxidase activity, further alleviating oxidative stress (Kim et al., 2011). Additionally, Nrf2α subunit was found to bind with sirtuin-3 (SIRT3) promoter directly, leading to the upregulation of SIRT3 expression. And the elevation of SIRT3 further activated SOD through promoting SOD deacetylation and then reduced mitochondrial oxidative stress (Satterstrom et al., 2015; He et al., 2019). Moreover, another possible mechanism by which melatonin suppresses oxidative stress involves increasing GSH content (Paterniti et al., 2016). Nrf2 can promote GSH biosynthesis enzymes and GSH reductase (GSR) expression, and thus exerts a valid effect in preserving the mitochondrial GSH pool (Ryoo and Kwak, 2018). High concentrations of GSH are oxidized to glutathione disulfide (GSSG) by GPx, thereby reducing peroxides to non-toxic substances and alleviating oxidative stress (Harvey et al., 2009).

In addition to activating Nrf2, studies have also demonstrated that administration with melatonin can play protective effects via inhibiting NADPH oxidase, which can produce ROS upon activation (Wilkinson and Landreth, 2006; Li et al., 2014). Specifically, in a rat MCAO/R model, by hindering phosphatidylinositol 3-kinase (PI3K)/Akt signaling pathway, melatonin can suppress the p47phox subunit phosphorylation, and thus block the combination of p47phox with gp91phox subunit. This process disturbs the assembly and activation of NADPH oxidase, ultimately alleviating oxidative stress (Li et al., 2014).

### 4.2 Neuroinflammation

Neuroinflammation is the inflammatory response concentrated in the CNS and might be initiated from ischemia. It is featured as the accumulation of pro-inflammatory factors, comprise IL-1β, IL-6, TNF-α and inducible nitric oxide synthase (iNOS) that are generated from activated microglia and astrocytes after ischemic stroke (Jin et al., 2010; DiSabato et al., 2016; Yang and Zhou, 2019). Melatonin can hinder microglia and astrocytes phenotype switching through multiple mechanisms, further attenuating neuroinflammation.

During the I/R process, two different forms of microglia are involved, including pro-inflammatory microglia (M1) and anti-inflammatory microglia (M2) phenotypes. Under the ischemic conditions, M2 phenotype transforms into the active M1 phenotype, which promotes further pro-inflammatory cytokines release and eventually accelerates stroke-induced secondary brain injury (Jin, Yang, and Li, 2010). The switch and balance between the M1/M2 phenotype can be modulated by melatonin through various mechanisms, including inhibiting nuclear factor kappa-B (NF-κΒ), activating signal transducer and activator of transcription 3 (STAT3) and interaction with MT2 receptor. Detailly, Zhao et al. demonstrated that in MCAO/R rats, melatonin could inhibit TLR4 expression, further hindering downstream NF-κΒ activation. NF-κΒ inactivation can initiate the transformation of microglia from M1 to M2 phenotype, thereby down-regulating the release of M1 phenotype-related pro-inflammatory factors (Y. Zhao et al., 2019; J. Sun et al., 2019; Ji et al., 2017). In addition, treatment with melatonin was also confirmed to significantly upregulate the expression of phosphorylated STAT3 in microglial cells (Z.J. Liu, Ran, et al., 2019). The phosphorylation of STAT3 has been demonstrated to exert a crucial effect in IL-10-induced anti-inflammatory effects. Specifically, removing the docking sites of STAT3 from IL-10R1 would render the receptor unable to transduce the signal for suppressing cytokine synthesis. Thus, the activated STAT3 may contribute to the anti-inflammatory cytokines expression which induces microglia transforming from M1 to M2 phenotype and further inhibits neuroinflammation (Williams et al., 2004). Besides, in a rat MCAO/R model, melatonin was found to act on MT2 and increase the ratio of triggering receptor expressed on myeloid cells 2 (TREM2)/iNOS, a marker for the transformation of M1 to M2, which eventually resulted in the suppression of neuroinflammation (Azedi et al., 2019).

Melatonin also exerts anti-inflammatory effect through suppressing the proliferation and activation of astrocytes. Regarding astrocytes, they are involved in BBB formation and maintenance, synaptogenesis, neurotransmission, and metabolic regulation. Under ischemic conditions, cytokines produced by activated microglia cause astrocyte reactivity hyperplasia and transform into neurotoxic reactive astrocytes, which further result in cerebral damage (Liu and Chopp, 2016; Liddelow et al., 2017; Yang et al., 2020). Chen et al. reported that administration with melatonin attenuated TRIF expression, an adapter molecule of TLRs, and ultimately prevented the conversion of astrocytes from anti-inflammatory (A2) to pro-inflammatory (A1) phenotype (Chen et al., 2020). In addition, in rats subjected to MCAO/R, melatonin administration could effectively decrease glial fibrillary acid protein (GFAP) expression, C3, and S100A10, suggesting that melatonin inhibited reactive astrogliosis and A1 astrocyte polarization (Yawoot et al., 2022). Besides, melatonin was also shown to reduce astrocyte-mediated inflammatory response by inhibiting glycogen synthase kinase-3 beta (GSK-3β) expression levels and receptor-interacting serine/threonine-protein 1 kinase (RIP1K) activities, consequently enhanced axonal regeneration and promoted neurobehavioral recovery (Yawoot et al., 2022). Mechanistically, suppression of GSK-3β can reduce NF-κB nuclear translocation and upregulate cyclic AMP response element-binding protein (CREB) transcription. These cause a relative increase in CREB compared to NF-κB in the nucleus, resulting in increased binding of CREB Binding Protein (CBP) with CREB and decreased interaction between CBP and NF-κB p65. The increased combination of CBP and CREB produces high levels of IL-10, a critical anti-inflammatory factor (Maixner and Weng, 2013). Meanwhile, the reduced interaction of CBP with NF-κB p65 inhibits the pro-inflammatory factors, and thus alleviates neuroinflammation (Martin et al., 2005).

### 4.3 Apoptosis

Apoptosis is an ATP-dependent death program characterized by fragmentation of chromosomal DNA, degradation of cytoskeletal, and generation of apoptotic bodies (Elmore, 2007; Xu et al., 2019). Regarding the apoptosis pathways, two major patterns are widely accepted including the extrinsic pathway and the intrinsic pathway. The extrinsic pathway refers to receptor-mediated apoptosis, and the activation of death receptors involves in two main ligands, including tumor necrosis factor (TNF) and Fas. Ischemia can induce the combination of the ligands with death receptors which lead to apoptosis and eventually cerebral injury. While in the intrinsic pathway, ischemia could directly induce the generation of intracellular signals and the subsequent formation of mitochondrial permeability transition pore, induce cytochrome C release into cytoplasm and activate caspase-3, and eventually lead to apoptosis (Broughton et al., 2009). In addition, members of Bcl-2 protein family, such as Bim, Bad, Bid, Bcl-x (L), and Bcl-2, are responsible for regulation of apoptosis via modifying the permeability of mitochondrial membrane (Cory and Adams, 2002; Elmore, 2007).

Previous studies have reported that melatonin could play anti-apoptotic functions during cerebral I/R injury through multiple biological mechanisms, including activation of the PI3K/Akt, Raf/MEK/extracellular-regulated kinase (ERK) signaling pathway as well as inactivation of Jun N-terminal kinases (JNK) pathway (Koh, 2008c). In 2008, Koh et al. elucidated that pretreatment with melatonin in the rat model of MCAO-induced cerebral ischemia could significantly reduce the infarct volume by activating Akt. Specifically, melatonin administration can promote the phosphorylation of specific residues on forkhead rhabdomyosarcoma transcription factors (FKHR) by activating PI3K/Akt. Then, 14-3-3 protein, an anti-apoptotic factor, would directly interact with the phosphorylated FKHR and anchor the phosphorylated FKHR within the cytoplasm. These FKHR are blocked from further translocation into the nucleus, therefore inhibiting downstream target genes transcription, including Fas ligand (Koh, 2008d). In 2021, Ran et al. elucidated that both in MCAO/R mice model and N2a cells exposed to OGD/R, treatment with melatonin could significantly reduce the brain infarct volume and neuronal cells apoptosis by activating Akt. Specifically, melatonin administration can decrease phosphatase and tensin homolog (PTEN) deleted on chromosome 10 activity through promoting its phosphorylation. PTEN is a major up-stream negative regulator of the PI3K/Akt signal transduction which can promote the dephosphorylation of PIP3. In the presence of melatonin, phosphorylated PTEN levels were increased, thereby decreasing PTEN activity and activating PI3K/Akt pathway (Ran et al., 2021). Similarly, in a MCAO/R mouse model, melatonin was shown to activate the phosphorylation of Akt at Thr308 via PI3K/pyruvate dehydrogenase kinase-1 (PDK-1) signaling, which further inactivate GSK-3α/β, two critical players in the activation of apoptosis (Kilic et al., 2017).

Besides, in the cerebral MCAO model, melatonin was found to increase the phosphorylation of pro-apoptotic protein Bad through activating PI3K/Akt, further preventing Bad from interacting with Bcl-x(L) and blocking the activation of downstream apoptosis pathway (Koh, 2008b).

Apart from activating PI3K/Akt pathway, Koh et al. also demonstrated that in a rat MCAO model, treatment with melatonin can protect brain tissue from ischemic injury through Raf/MEK/ERK signaling pathway by modulating the pro-apoptotic protein phosphorylation. To some extent, melatonin can increase the phosphorylation of Raf-1 as well as MEK. And the activation of these two proteins kinases can phosphorylate ERK1/2 which results in the phosphorylation of its downstream target 90 ribosomal S6 kinase (90RSK) (Koh, 2008a). The phosphorylated 90RSK may further phosphorylate the Ser112 residue of Bad and inactivate the pro-apoptotic effect of Bad (Zha et al., 1996; Koh, 2008a). Moreover, Chen et al. reported that melatonin can effectively suppress fork-head box O3a (FoxO3a) activity by inhibiting JNK, thereby hindering the binding of FoxO3a to Bim promoters and the subsequent expression of Bim proapoptotic protein (Chen et al., 2022). Besides, JNK has been shown to regulate cytochrome C release from mitochondria through inducing Bid cleavage. Melatonin could attenuate JNK activation, thereby hindering cytochrome C release and suppressing cytochrome C-induced apoptosis cascades (Madesh et al., 2002; Song et al., 2014).

### 4.4 Autophagy

Autophagy is a lysosome-mediated self-digestion and recycling process that contributes to clearing folded or aggregated proteins and promoting the degradation of damaged organelles. It plays a key role in preserving cellular homeostasis under physiological conditions (Glick et al., 2010; Boga et al., 2019). However, under pathological circumstances including cerebral I/R injury, ischemia-mediated excessive autophagy activation may be harmful and further lead to cell death. Autophagy begins with nucleation of the phagophore induced by the unc-51-like autophagy activating kinase 1/2 (ULK1/2) complex. Then, the phagophore elongates with the aid of autophagy-related (ATG) proteins and finally the expanding membrane forms an autophagosome which closes around its cargo. The outer membrane of the autophagosome subsequently fuses with lysosomal membrane, and further generate an autolysosome, lead to autophagic cargo degradation (Itakura and Mizushima, 2010; Wong et al., 2013; Parzych and Klionsky, 2014).

Accumulating evidences have demonstrated that melatonin could promote physiological autophagy, while inhibiting excessive autophagy to exert neuroprotective effects. On the one hand, in hippocampal HT22 cells exposed to OGD/R, melatonin was shown to increase the expression of both Ras-related protein 7 (Rab7) and transcription factor Forkhead box class O1 (FoxO1), subsequently promoting autophagosome maturation and attenuating ischemic-like injury (Luchetti et al., 2022). Furthermore, both in a MCAO/R model and HT22 cell line exposed to OGD/R, treatment with melatonin upregulated SIRT1, a histone deacetylase that can cause the deacetylation of brain and muscle ARNT-like protein 1 (BMAL1) and decrease its degradation. The upregulation of BMAL1 could promote the expression of ATG14 through directly binding to the E-box elements in the ATG14 promoter, subsequently increasing the level of Beclin-1 and the ratio of LC3II/LC3I, enhancing autophagy and improving neurological function (Liu et al., 2021). On the other hand, melatonin could activate mammalian target of rapamycin complex1 (mTORC1) through multiple pathways, thereby hindering excessive autophagy and exerting neuroprotective effects. Specifically, in a rat MCAO/R model, melatonin was observed to decrease the phosphorylation of tuberous sclerosis complex 2 (TSC2) through PI3K/Akt signaling pathway. Therefore, the downstream protein complex TSC1/TSC2 was disrupted, leading to stabilization of the downstream Rheb-GTPase and subsequent activation of mTORC1 (Yang and Klionsky, 2010; Zheng et al., 2014). The mTORC1 can inactivate the autophagy initiators ATG13 and ULK1/2 through combination and phosphorylation, regulate the class III PI3K complex, and thus exerts an important effect in inhibiting autophagy (Rabanal-Ruiz, Otten, and Korolchuk, 2017). Likewise, mTORC1 can also phosphorylate transcription factor EB (TFEB) and TFE3 which facilitates the interaction between these two transcription factors and cytosolic chaperone 14-3-3 and retains them in cytoplasm (Martina et al., 2012). Therefore, the expression of several autophagy-related genes including PIK3C3 and ATG16L1, etc., are inhibited, thereby suppressing excessive autophagy and reducing cerebral injury (Settembre et al., 2011).

In addition to activating mTORC1 signaling pathway, melatonin could also attenuate excessive autophagy through hindering JNK signaling pathway, according to previous studies. Melatonin can inactivate JNK1, which further inhibits the separation of Bcl-2 from Beclin-1. Beclin-1 acts as a part of class III PI3K complex, and the combination of Bcl-2 and Beclin-1 can hinder class III PI3K complex activation, thereby inhibiting autophagy (H.D. Xu and Qin, 2019; Zheng et al., 2014). Similarly, in a rat MCAO/R model, Feng et al. reported that pretreatment with melatonin could inhibit pancreatic ER kinase (PKR)-like kinase (PERK)/Inositol-requiring enzyme 1 (IRE1) pathway, further suppressing the activation of the JNK, subsequently preventing the uncoupling of Bcl-2 with Beclin-1 and excessive autophagy (Feng et al., 2017). Moreover, in a rat MCAO/R model and PC12 cell OGD/R model, pretreatment with melatonin was shown to inhibit excessive autophagy through upregulating miR-26a-5p expression and then downregulating downstream neuron-restrictive silence factor (NRSF) expression (Yang et al., 2020).

### 4.5 Glutamate excitotoxicity

Glutamate acts as the predominant excitatory neurotransmitter in the CNS (Teichberg et al., 2009). During cerebral I/R injury, ischemia can lead to the excessive glutamate release, which triggers the rapid influx of calcium into the cell cytoplasm through the overstimulation of N-methyl-D-aspartate receptors (NMDARs), thereby resulting in a succession of harmful signaling cascades and subsequent CNS cells death (Tehse and Taghibiglou, 2019; M. Zhou and Baudry, 2006; Sattler and Tymianski, 2000). Moreover, glutamate can also cause the overproduction of nitric oxide (NO) and the decrease of downstream mediator protein phosphatase 2A (PP2A) which result in neurotoxicity and cerebral injury (Koh, 2012a; Jia et al., 2015). Melatonin can reduce glutamate excitotoxicity during cerebral I/R injury through modulating glutamate as well as its receptor pathways.

Detailly, Patino et al. confirmed that in rat hippocampal slices exposed to OGD/R, melatonin could directly reduce the release of glutamate and prevent a persistent activation of NMDARs, thereby reducing intracellular Ca^2+^ levels (Patino et al., 2016). Similarly, in hippocampal slices exposed to OGD/R, melatonin reduced a surge of synaptic glutamate release and neuronal cell death after ischemia-reperfusion, which further strongly suppressed the increase in the intracellular Ca^2+^ concentration by downregulating the NMDAR activity (Furuta et al., 2022). Besides, in hippocampal HT22 cell lines exposed to glutamate excitotoxicity, melatonin pretreatment could prevent glutamate-mediated reduction in calcium-buffering proteins such as parvalbumin and hippocalcin and thus downregulate Ca^2+^ levels (Koh, 2012b). Similarly, in Sprague-Dawley rat model subjected to MCAO, melatonin has also been documented to reduce intracellular Ca^2+^ levels through increasing the expression of parvalbumin and hippocalcin (Koh, 2012b). Additionally, melatonin can directly bind with calreticulin and further reduce the level of Ca^2+^ by hindering the release of Ca^2+^ from endoplasmic reticulum, in rat C6 astroglial cells exposed to glutamate excitotoxicity (Das et al., 2008; Venkatesan et al., 2021). With the decrease of Ca^2+^ levels, calpain expression and caspase-3 activation are inhibited. Calpain inhibition subsequently reduces the degradation of cytoskeletal proteins as well as axon-myelin structural unit, thus maintaining the structural integrity of CNS cells and preventing glutamate excitotoxicity (Samantaray et al., 2008; Doshi and Lynch, 2009).

Moreover, in a MCAO rat model, melatonin was found to interact with calmodulin, further preventing the binding of calmodulin to NOS, and thus inhibited calmodulin-dependent NOS activation (Koh, 2008e). Apart from inhibiting NOS activity, Koh et al. suggested that in a rat focal cerebral ischemia model, treatment with melatonin could mitigate glutamate-induced decrease of PP2A subunit B, a critical subunit in facilitating various functions of PP2A. As an essential protein phosphatase, PP2A can promote DNA repair, cellular proliferation and differentiation. From this point of view, melatonin can maintain PP2A levels, thereby promoting neuronal survival and alleviating glutamate excitotoxicity (Koh, 2012a).

### 4.6 Mitochondrial dysfunction

Mitochondria serve as essential organelles that exert a crucial effect in maintaining energy metabolism and cellular homeostasis (Anderson and Sims, 1999; Lesnefsky et al., 2017). Under normal circumstances, mitochondria produce ATP through the electron transport in the respiratory chain to keep cellular function and integrity (Newmeyer and Ferguson-Miller, 2003). Meanwhile, mitochondria could release pro-apoptotic factors, and thus control cell survival (Ott et al., 2002). However, under pathological conditions such as cerebral I/R injury, ischemia can lead to mitochondrial dysfunction, including impaired ability to generate ATP, accumulation of ROS, transition of the mitochondrial permeability and increased release of pro-apoptotic factors (Sims and Anderson, 2002; Ham and Raju, 2017). Melatonin has been found to play neuroprotective effects by improving ischemia-induced disturbance in mitochondrial redox state, fusion and fission, biogenesis, mitophagy and mitochondrial transfer.

Firstly, as a potent free radical scavenger, melatonin has the capacity to directly counteract mitochondrial oxidative injury. In a MCAO/R mice model, melatonin pretreatment significantly increased SIRT1 expression and thus reduced the expression of acetylated p53 and NF-κB. Subsequently, the deacetylated p53 and NF-κB maintained mitochondrial membrane potential, elevated the activity of mitochondrial Complex I and reduced mitochondrial ROS levels (Yang et al., 2015). Apart from alleviating mitochondrial oxidative damage, in N2a neuroblastoma cells exposed to OGD/R as well as a MCAO/R rat model, melatonin was shown to upregulate OPA1 expression, one of the mitochondrial fusion-related proteins, by activating Hippo/Yap pathway. Then, the mitochondrial cristae junctions are tightened and cytochrome c release is restricted, thereby improving OPA1-related mitochondrial fusion and inhibiting mitochondrial fission (Wei et al., 2019; Nasoni et al., 2021). Moreover, Nasoni et al. reported that in hippocampal HT22 cells with excessive mitochondrial oxidative stress, melatonin was found to enhance the levels of peroxisome proliferator-activated receptor gamma coactivator 1 alpha (PGC-1α) expression. Subsequently, PGC-1α can further enhance the combination of estrogen-related receptor (ERR) α with ERR binding element. Thus, SIRT3 expression is initiated which further induces mitochondrial biogenesis through activating nuclear respiratory factors-1 (NRF-1) and nuclear respiratory factors-2 (NRF-2) and subsequently increasing expression of mitochondrial transcription factor A (mtTFA) (Kong et al., 2010; Nasoni et al., 2021). Besides, in HT22 cells exposed to OGD/R, melatonin was shown to promote mitochondrial transfer and reshape mitochondrial network via increasing the number of tunneling nanotubes (TNTs). TNTs are dynamic structures that connect cells, and intercellular mitochondria could transfer through these structures, which further compensate for damaged organelles and promote cell recovery (Nasoni et al., 2021). Lastly, in HT22 neuronal cell line with glutamate injury, melatonin could upregulate the levels of Bcl-2 expression and downregulate the levels of Beclin-1 expression, thereby further reducing glutamate-induced mitophagy and restoring mitochondrial function (Wang et al., 2019). In addition, in PC12 cell line treated with ropivacaine, melatonin was shown to reduce the expression of two mitophagy-related proteins, PTEN-induced kinase I (PINK1) and Parkin, which may hinder the occurrence of mitophagy (Yu et al., 2018; Wang et al., 2019; Zeng et al., 2022).

## 5 Clinical prospects

In recent years, numerous clinical investigations exploring the influence of melatonin on ischemic stroke and the sequelae of ischemic stroke have been reported. Firstly, a series of preclinical studies have shown melatonin was an extremely safe neurotherapeutic agent even at high concentrations (Romero et al., 2016). So far, melatonin has been applied to treat a variety of diseases, involving sleep disorders, depression, etc., and no clinical studies on melatonin therapy have shown serious side effects (Cardinali et al., 2012; Hassell et al., 2015; Riha, 2018). Secondly, pineal calcifications diagnosed by applying multi-spiral computer and/or magnetic resonance tomography has been described as having a significant impact on the risk of ischemic stroke (Kitkhuandee et al., 2014). Besides, subsequent studies confirmed a positive relationship between pineal calcifications and decrease of melatonin synthesis (Schmid et al., 1994). Thus, human clinical trials of ischemic stroke to evaluate potential clinical applications of melatonin seemed to be necessary. Current studies have found that endogenous melatonin, mainly produced by the pineal gland, might be a good predictor for the prognosis of ischemic stroke. In a prospective observational study published in 2018, Lorente et al. found that in patients with severe middle cerebral artery infarction, serum melatonin concentrations were associated with total antioxidant capacity as well as malondialdehyde levels (used to assess lipid peroxidation). Furthermore, results of this research also illustrated a positive correlation between serum melatonin levels and the severity or even mortality of stroke patients during the 30-day follow-up period (Lorente et al., 2018). For this point of view, we speculated that patients with more severe ischemic stroke would produce high levels of ROS, which might result in higher concentrations of serum melatonin to compensate for the increase in oxidation products. Among them, when the attempts to maintain a balance between oxidation and antioxidant status were insufficient, the patients with severe ischemic stroke would eventually die. Additionally, a case-control study involving 42 patients with ischemic stroke demonstrated that the levels of melatonin in the urine was remarkably decreased in contrast to the control value. It was reasonable to suspect that due to the excessive free radical production, the catabolism of melatonin might be speeded up during acute ischemic stroke, indicating the vital role of melatonin in scavenging free radicals and neuroprotection (Ritzenthaler et al., 2013).

A previous systematic review focusing on clinical trials of melatonin on various brain injury provided evidence that melatonin treatment improved sleep disturbance following traumatic brain injury and increased survival rate in intubated patients with hemorrhagic stroke and asphyxiated newborns (Ramos et al., 2020). Regarding the clinical potential of exogenous melatonin therapy on cerebral I/R injury and ischemic stroke, a double-blind randomized controlled trial recruited 60 patients, who orally received melatonin at 6 mg/day for 3 days before and after carotid endarterectomy, to explore the therapeutic effect of melatonin on cerebral I/R injury (Yang et al., 2018). The circulating levels of inflammatory cytokines in patients of the melatonin group were significantly reduced, implying that melatonin could decrease the inflammatory damage and ameliorate subsequent brain I/R injury. Furthermore, Mehrpooya et al. recruited 65 patients with acute ischemic stroke who were not eligible for reperfusion treatment in a double-blind placebo-controlled trial. They observed that melatonin supplementation within 24 h after stroke at a dose of 20 mg once daily for 5 days had a higher reduction in modified Rankin Scale score (mRS) at 30 and 90 days after treatment, indicating melatonin had beneficial effects on neurological recovery after ischemic stroke (Mehrpooya et al., 2022). As one of the holy grails for acute ischemic stroke treatment, neuroprotection could theoretically improve the neurological disability of stroke survivors, which makes it attract widespread attention. According to previous studies, therapeutic hypothermia can maintain organ vitality and is one of the most powerful neuroprotective treatments (Perlman et al., 2010; Kurisu and Yenari, 2018). Although no clinical researches involving in the neuroprotective effect of melatonin combined with hypothermic therapy in cerebral I/R injury treatment following ischemic stroke, a clinical trial recruiting 30 newborns with confirmed hypoxic-ischemic encephalopathy demonstrated that early use of melatonin combined with hypothermia could lower plasma free radicals generation, protect against the subsequent CNS injury and ultimately improve survival rate of patients (Aly et al., 2015). Additionally, as a common and severe complication of ischemic stroke, poststroke delirium (PSD) has attracted wide attention from researchers due to it was related to increased mortality, longer hospital stays, and lower functional outcome (Siddiqi et al., 2006; Shaw et al., 2019). A propensity score-matched analysis involving 573 patients with acute ischemic stroke showed that patients who received prophylactic treatment with melatonin within 24 h after stroke onset had a lower risk of PSD compared with patients receiving standard treatment (Mengel et al., 2021). Although the underlying mechanism of melatonin for preventing PSD was still unclear, the evidence of this propensity score-matched analysis implied that supplementation with melatonin could reduce PSD susceptibility, and further supported the neuroprotective effect of melatonin on acute ischemic stroke. Taken together, recent clinical studies have partially clarified the predictive and therapeutic potential of melatonin for ischemic stroke. Nevertheless, further studies, ideally enrolling more clinical subjects, are still needed to evaluate the clinical safety and effectiveness of melatonin to better understand its medical effects in cerebral I/R injury and ischemic stroke.

## 6 Conclusion

In the past decades, ischemic stroke has been one of the most threatening diseases for human health. Due to its high morbidity and mortality, efforts have been made for detecting appropriate alternative or complementary drugs on ischemic stroke therapy. Currently, accumulated lines of evidence suggested that melatonin could reduce cell death and increase cell viability, and display an obvious neuroprotective effect on various CNS cells *in vitro*. Meanwhile, experimental stroke models *in vivo* have confirmed that melatonin would reduce infarct size and brain edema, and improve neurological function. Subsequently, we also outlined the extensive studies and performed a comprehensive introduction of various functions of melatonin in cerebral I/R injury, including regulating oxidative stress, neuroinflammation, apoptosis, autophagy, glutamate excitotoxicity and mitochondrial dysfunction. Meanwhile, some key signaling pathways has been demonstrated to play important roles in melatonin neuroprotection, such as Nrf2, NF-κΒ, PI3K/Akt, Raf/MEK/ERK, JNK, and Hippo/Yap pathways. In future studies, the biomolecular mechanism of melatonin in cerebral I/R damage still needs in-depth research, which is necessary for its clinical application in ischemic stroke.

Given its multiple roles against cerebral I/R damage, it is not surprisingly that melatonin has been considered as a promising candidate, both as a diagnostic biomarker for stroke prognosis and as a drug target for stroke therapy. Recent studies have demonstrated a potential relationship between circulating melatonin levels and the severity and mortality of stroke patients. Regarding exogenous melatonin therapy, oral melatonin in patients undergoing carotid endarterectomy could reduce serum pro-inflammatory cytokines levels and alleviate cerebral I/R injury to a certain extent. Moreover, early treatment with melatonin following stroke may help improve functional recovery in patients ineligible for reperfusion therapy. However, there are still some limitations to the clinical application of melatonin, and some potential issues should be considered in future clinical trials. First, high-quality randomized controlled trials with larger sample sizes and more ethnic groups are needed to further explore the clinical efficacy and side effects of melatonin in patients with ischemic stroke. Second, inappropriate treatment time windows, insufficient dosage or treatment duration may be responsible for the lack of positive results in clinical trials. Thus, the dosage, timing, and duration of melatonin require further reconsideration to determine the optimal application strategy in stroke patients. Third, melatonin might be more effective when combined with other types of stroke treatments, such as hypothermia or hyperbaric oxygen therapy, and the effectiveness of combination therapy should be fully investigated in the future.

In summary, based on the information reviewed above, as a low toxicity and well-tolerated agent, melatonin supplement was supposed to be a new and prospective treatment method for cerebral I/R injury. More randomized and multiple-center clinical trials were need to be designed to confirm predictive and therapeutic role of melatonin in ischemic stroke.
